# Analysis of minimal complex systems and complex problem solving require different forms of causal cognition

**DOI:** 10.3389/fpsyg.2014.00739

**Published:** 2014-07-16

**Authors:** Joachim Funke

**Affiliations:** Department of Psychology, Experimental and Theoretical Psychology, Ruprecht-Karls-Universität HeidelbergHeidelberg, Germany

**Keywords:** problem solving, causality, dynamic decision making, strategies, knowledge

In the last 20 years, a stream of research emerged under the label of “complex problem solving” (CPS; see e.g., the two editions from Frensch and Funke, 1995 and Sternberg and Frensch, 1991). This research was intended to describe the way people deal with complex, dynamic, and intransparent situations. One of the promoters of this field, Dietrich Dörner from Bamberg University, proposed to use complex computer-simulated scenarios as stimulus material in psychological experiments (see e.g., Brehmer and Dörner, 1993). This line of research lead to subtle insights into the way how people deal with complexity and uncertainty (see Dörner, 1997; Osman, 2010).

Besides knowledge-rich, realistic, intransparent, complex, dynamic scenarios with many variables, a second line of research used more simple, knowledge-lean scenarios with a low number of variables (“minimal complex systems,” MCS) that have been proposed recently in problem-solving research for the purpose of educational assessment (see Greiff et al., 2012). In both cases, the idea behind the use of microworlds is to increase validity of problem solving tasks by presenting interactive environments that can be explored and controlled by participants while pursuing certain action goals.

The construction principles behind the minimal complex systems follow certain formalisms like linear structural equations or finite state automata (both described in Funke, 2001). Subjects have to first explore such systems (they have to understand the causal relations between input and output variables) and then use the acquired causal knowledge to control the given system in order to reach given goal values.

The main argument presented here is: both types of systems—CPS and MCS—can only be dealt with successfully if causal dependencies between input and output variables are identified and used for system control. System knowledge is necessary for control and intervention. But CPS and MCS differ in their way of how causal dependencies are identified and how the mental model is constructed; therefore, they cannot be compared directly to each other with respect to the cognitive processes that are necessary for solving the tasks.

The argument in more detail: In case of the more simple MCS problems, a complete causal analysis of the system under scrutiny can be done in short time (e.g., in 3 min). Typically, the acquired causal knowledge is assessed via a causal diagram that has to be drawn by the participant (Blech and Funke, 2006). In case of the more complex CPS systems, time is not enough to run a complete causal analysis of the given scenario because of its complexity. Normally, causal knowledge about the CPS system is not assessed (there are exceptions: e.g., Wittmann and Süß, 1999). Instead, the use of heuristics and the use of causal knowledge derived from previous everyday experience are necessary for constructing a causal model.

So, the role of causal cognition is different in both types of problems. In the simpler knowledge-lean systems, systematic causal analysis is the main task during exploration; no reliance upon previous knowledge (except from strategic knowledge) is recommended. A detailed point-by-point analysis is needed, domain knowledge is not important at all (instructions sometimes warn to rely on such knowledge). In the more complex knowledge-rich systems, a precise causal analysis through systematically controlled exploration is nearly impossible; instead, reliance upon previous content knowledge is highly important.

In the next section, I will contrast the two approaches by describing the role of causal analyses for a problem-solver who has to handle either the task of carefully analyzing systems from the MCS type or systems from the CPS type.

## MCS: problem situations that could be understood completely by precise causal analyses

**Figure 1:**
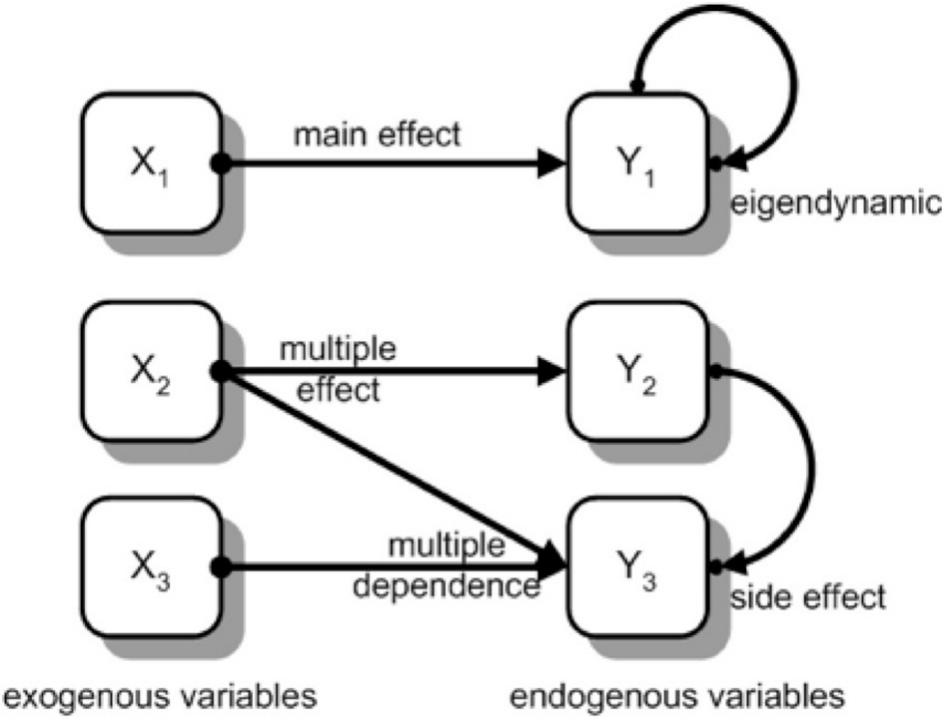
**A typical MicroDYN item as an example for a more simple system with different kinds of effects**. For the selected sets of endogenous and exogenous variables any cover story is possible (from Greiff et al., 2012, p. 192).

Problems that could be understood completely by a precise causal analysis need to consist of a small set of input and output variables. Otherwise, constraints of working memory and of time for analysis would make such a complete analysis impossible. For problem-solving research within educational contexts, Greiff et al. (2012) proposed the use of MicroDYN items according to the formalisms of linear structural equations (see Funke, 2001) that could be analyzed within 5 min testing time. A typical MicroDYN example containing all possible types of effects between input and output variables is shown in Figure 1.

The task for the subject is twofold: first, to explore the system by manipulating the input variables and observing the effects of this manipulation on the output variables; second, to control the output variables with respect to given goal values by appropriate manipulation of the input variables.

As one can easily see, this task requires nothing else than identifying causal structures by active experimentation and using the identified causal structures later for reaching goals. The task is designed so that subjects have a chance to identify all causal relations by proper variation of the inputs. No wonder that the VOTAT strategy (“Vary One Thing At a Time”; Tschirgi, 1980) is seen as most relevant for succeeding with the task (Fischer et al., 2012).

An advantage of this type of causal structure is its independence from content: There is a nearly arbitrary choice of labeling input and output variables and a nearly arbitrary choice of selection for the type of relationship (see Figure 1: main effects; multiple effects; multiple dependencies; side effects; eigendynamic). So, complexity of the item structure can be changed easily to construct large sets of items with different difficulties (as it is needed, e.g., in large-scale assessments like the OECD “Programme for International Student Assessment”).

The disadvantage of this procedure is the unclear degree of overlap between previous knowledge about the assumed relationships between the variables (on the side of the participant who has to identify the causal structure) and the realized relationships. So, different item difficulties result not only as a consequence of the complexity of the chosen causal structure, but also of the (unknown) degree of “surprise” to the participant. To decrease this potential disadvantage, item labeling is very unspecific (“variable A,” “controller 2,” etc.), loosing connection to everyday knowledge.

## CPS: problems that could not be understood completely by precise causal analyses

Problems that could not be understood completely by precise causal analyses consist of a larger number of variables that cannot be analyzed completely in a given time frame of about an hour in the lab that is given for (short) exploration and (longer) control of the system. As an example take the microworld “Tailorshop” (originally developed by Dietrich Dörner). The round-driven scenario simulates a small business that produces and sells shirts. The participants lead this business for 12 simulated months by manipulating several variables like the number of workers, the expenses for advertising, etc. (see Figure 2 from Danner et al., 2011, on p. 226, for the complete set of variables; this list is normally not shown to the participants).

The task for the subject is to increase the company value over the course of the simulation period. Participants have to rely on assumed causal relations but cannot check the details in this case. They have to monitor the systems' output in a more global way than in a MCS situation. The famous VOTAT strategy that is helpful in the previously mentioned MCS example would not work in this situation because there are too many variables that could not be controlled for in short time. This is a typical situation for many everyday complex problems: we cannot use VOTAT alone to find out how to increase the quality of the relationship with our partner; policy-maker cannot systematically change the conditions of a nations' education system in order to find the most efficient one.

How can we deal with such complex situations, anyway? If one learns about the variables of the Tailorshop, a participant might hypothesize that “workers' satisfaction” is more dependent on “salary” than from “social costs” or that “price of shirts” has more influence on “demand” than “number of shops” In real life as well as in CPS simulations, general knowledge about the world and knowledge about the domain in question is guiding our problem solving activities. It is important how variables are labeled semantically: because, for sure, the “Machine 100” produces double the amount of shirts than “Machine 50”—the knowledge about these labels guides decision-making much more than any systematic identification strategy.

## On the universality of causal cognition and on the cultural specificity of heuristics

For influencing the world, the assumed causal mechanisms (in the case of CPS, e.g., “more salary for workers increases their satisfaction”; in the case of MCS, e.g., “increasing controller 1 by a value of 2 decreases variable C by the value of 8”) remain the same but the reliability of the rule is lower in the case of CPS (How much does satisfaction increase if one increases salary from 900 to 1000 units? Is it a linear function? Has this function an upper limit? Does the relation depend on other variables?) than in the case of MCS. The mechanism for producing/activating this causal knowledge is different: in the case of MCS, it is systematic experimentation and testing, in the case of CPS it is hope that some unproved world knowledge might apply to the given case.

There is some research on cultural differences in dealing with complexity and uncertainty. Strohschneider and Güss (1999) compared students from India and Germany while working on the complex scenario “MORO.” Subjects in this scenario had to take the role of a developmental aide and to improve the living conditions of the Moro tribe sustainably over a period of 20 (simulated) years. In their conclusions about the detected cultural differences, Strohschneider and Güss (1999, p. 250) state: “We thus explain the problem-solving differences by differences in strategic (or heuristic) expertise, and we argue that these differences in expertise are due to a number of specific characteristics of the cultural learning environment.”

On a more theoretical level, Medin et al. (2014; see also Medin and Atran, 2004) argue that cognition occurs in “cultural ecosystems”—it would be interesting to learn if even abstract MCS tasks would be conceptualized differently in different cultures. For CPS tasks, this happens for sure. Strohschneider and Güss (1998, p. 713), for example, observed differences in planning and problem-solving styles between German and Brazilian students with the MCS simulation “Coldstorage” that can be interpreted as “effects of different sociocultural conditions, such as accountability of the environment, value systems, and objective planning necessities.”

All organisms that have intentions and follow goals will, at some point in time, face problems in reaching them. For solving these problems, the obstacles between given and goal state need to be removed, and this presupposes causal cognition. Causal cognition (in the sense of understanding of causal relationships between input and output variables of dynamic systems) lies at the heart of problem solving when dynamic problems require the manipulation of exogenous variables in order to reach certain goal values in the endogenous variables.

When it comes to complex and knowledge-rich problems the use of heuristic decision rules is necessary; more important: the role of general world knowledge and of specific domain knowledge increases strongly. Therefore, cultural differences (Güss and Robinson, 2014) and cultural ecosystems (Medin et al., 2014) will become more visible when dealing with complex problems.

### Conflict of interest statement

The author declares that the research was conducted in the absence of any commercial or financial relationships that could be construed as a potential conflict of interest.
